# Popperian Dogs—Practical Rationality and Inferential Reasoning in Dogs

**DOI:** 10.3390/ani16060877

**Published:** 2026-03-11

**Authors:** Ludwig Huber

**Affiliations:** 1 Cognition and Applied Ethology, Messerli Research Institute, University of Veterinary Medicine, Veterinärplatz 1, 1210 Vienna, Austria; ludwig.huber@vetmeduni.ac.at; Tel.: +43-664-60257-6250; 2 Medical University of Vienna, 1090 Vienna, Austria; 3 University of Vienna, 1010 Vienna, Austria

**Keywords:** dogs, Popperian animal, practical rationality, inference by exclusion, selective imitation, theory of mind

## Abstract

The last few decades have provided mounting evidence that dogs are not only able to behave in intelligent ways but also that they have the capacity to make inferences by associating visible and imagined events. In particular, the kinds of inferences shown by dogs elevate them to ‘Popperian animals’, which are rational in the sense of being able to conduct trials and errors in the head. Here, I review a selection of studies with dogs that fulfil this attribution. Dogs have been found capable of (a) making inferences by exclusion, with both computer images and word-object associations, (b) drawing inferences about the efficiency of the observed methods, the situational constraints of the situation, and the goals of the model in the course of selective (‘rational’) imitation, and (c) going beyond directly observable cues when inferring the intention, the knowledge, or even the beliefs of humans in perspective-taking tasks. Despite the contamination with ambiguous data, open questions, and current debates about the possession of (non-linguistic) mechanisms for creating mental representations of the mental states of others (called mind reading), we may safely assume that domesticated dogs have become Popperian animals equipped with the cognitive tool set of inferential thinking and perspective taking.

## Simple Summary

Many people wonder whether dogs simply react to what they see and hear, or whether they can also “think ahead” and draw conclusions in their minds. This article reviews research showing that pet dogs can do more than follow simple habits: they can rule out wrong options to find the right one, copy others selectively when it seems the most efficient choice, and use clues to figure out what a person wants, knows, or mistakenly believes—even when those clues are subtle or out of sight. For example, dogs could choose a hidden treat by inferring where a person could or could not have seen it, and in another task, they adjusted their stealing when a person might be nearby but unseen, guided only by a sound they had learned to associate with that person. While some findings are mixed and not all dogs perform the same, the overall picture suggests dogs can run “mental trials” before acting. Understanding this richer dog mind can improve training, strengthen human–dog relationships, enhance welfare, and inform how we design supportive environments and roles for dogs in society.

## 1. Introduction

Although humans sometimes act unreasonably, we still consider the cognitive power of *Homo sapiens* superior to all other creatures on this planet. Aristotle already made a preliminary decision in this regard because he meant only man in possession of reason (*ratio*) and understanding (*intellectus*). This prejudice was the subject of many controversies among philosophers since then but in general survived to this day, called the “anthropological difference” [1]. Meanwhile, two streams of research challenge this. On the one hand, it is the paradigm shift in the philosophy of rationality and logic concerning human decision-making; on the other hand, it is the accumulating evidence of sophisticated decision processes in non-human animals.

Rationality is said to be “bound” because it is exercised by actors with limited capabilities and because it is far from being a tool for optimization or maximization [2]. Instead, it serves primarily to satisfy the actor’s expected utility by selecting those choices that are good enough, but not necessarily perfect, according to his or her desired outcomes. Such moderate conceptions of rationality leave room for widely observed phenomena of suboptimal human thinking. Rather than being discarded as “unintelligent behaviour”, these psychologically more realistic models of human decision-making can explain the countless deviations from perfect rationality as a symptom of the fact that we use formally imperfect but often more efficient “fast and frugal” heuristic procedures in everyday life [3].

Are the decision processes of non-human animals also of this fast and frugal manner? Or are all, even the seemingly “intelligent” and close-to-human-thinking performances of non-human creatures, simply attributable to genetically predetermined (automatic) behaviours plus some learning? Are we dealing with animal cognition only with innate or learned abilities instead of reason? Are even the decisions and actions of animals, which in humans are unhesitatingly explained by logical, deductive reasoning, also only a consequence of associative learning? A pure matter of trial and error?

**Figure 1:**
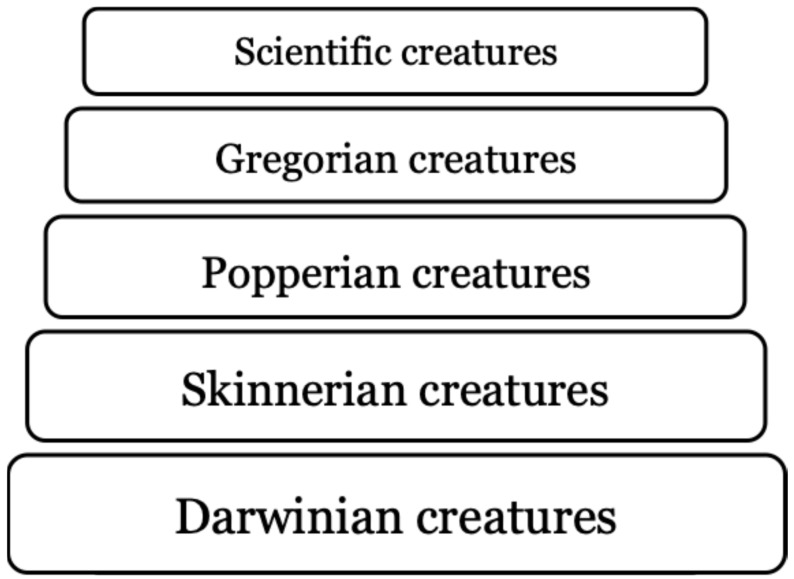
The tower of generate-and-test. Philosopher Daniel C. Dennett [5,6] proposed five hypothetical creatures arising from Darwin’s evolutionary process. Each of them uses generate and test but the process becomes more sophisticated with evolution. The progression runs from blind selection (Darwinian) to feedback-based learning (Skinnerian), to mental preselection (Popperian), to tool- and culture-amplified intelligence (Gregorian), culminating in science as the most powerful, systematic way to generate and test solutions.

But trial and error learning, commonly conceived as testing various possibilities and often defined as making mistakes until a successful solution is found and then repeating it and discarding ineffective approaches, is sometimes a dangerous undertaking. Missteps can be fatal. Therefore, the Viennese philosopher Karl Popper [4] propagated a different kind of this learning process, trial and error in thought, which is quicker and safer than operant conditioning, because it is better to let one’s hypotheses die in one’s stead. In reference to Popper’s concept of reasoning, of thinking hypothetically, and of considering possibilities without yet fully believing or intending them, philosopher Daniel Dennett called creatures with this ability “Popperian animals” [5,6] (Figure 1). These creatures possess cognitive, instrumental reasoning abilities to simulate different responses offline, evaluate their consequences, and discard bad options in advance. A Popperian animal is an organism capable of using internal representational models to test potential behaviours “offline”, allowing it to avoid dangerous or unproductive actions in reality. It discovers means by which to fulfil its purposes by trial and error with inner representations. According to Millikan [7], one of many reasonable interpretations of what it is to be rational is that being rational is being a Popperian animal. Still, one cognitive mechanism does not necessarily exclude the other, as associative learning and inferential capacities might interact and intermingle in different ways in the minds of Popperian animals [8].

## 2. Can Dogs Reason by Exclusion?

An often-used example of logical reasoning is transitive inference, which ultimately goes back to Aristotelian logic. If Rudolf is greater than Klaus and the latter in turn is greater than Robert, who is greater, Rudolf or Robert? The Swiss developmental psychologist Jean Piaget did not find this ability of basal logical thinking in children between two and seven years of age, but only in children between seven and 11 years of age, which he called the stage of “concrete-operational intelligence” [9]. At younger ages, children can infer inductively, i.e., generalize from individual cases, but they are not able to deductively infer the logically compelling consequences from given premises. A non-linguistic task to test transitive inference has been developed by comparative cognition researchers who replaced pairs of premises (or propositions) with successive discrimination tasks [10]. They trained squirrel monkeys sequentially with four stimulus pairs: A+ B−, B+ C−, C+ D−, and D+ E−. The letters represent different arbitrary stimuli, and the signs indicate whether the choice of stimulus in each pairing was rewarded (+) or not rewarded (−). In the crucial test (on the understanding of transitivity), the animals were presented with the novel pair B D. Although the two stimuli were never presented together before and were only together with other stimuli once rewarded and not once rewarded, the animals almost always chose stimulus B. The authors interpreted the preference for B over D in their monkeys as forming a linear representation of the pattern series and as consistent with the principle of transitive logic. Later, similar performances were found in chimpanzees, but also in rats and pigeons, as reviewed in [11], and even by baby domestic chickens [12]. Even more compelling and ecologically valid was the performance of Pinyon jays that applied the transitive logic to the assessment of dominance relationships between members of other groups after they could observe interactions between them [13].

Inferential processes are proper examples of the distinction between learning and thinking. One can distinguish the two processes by the type of information that the individual associates with. Whereas learning involves the association of two perceptual events, thinking is based on the association of a perceptual and an imaginary event—that is, a perceptually unavailable event [14]. Accordingly, the task of learning is to identify regularities of either purely external events (classical or Pavlovian conditioning) or associations between one’s own actions and external events (instrumental or Thorndikean conditioning). In contrast, reasoning can be understood as the search for the causes of events that cannot be explained and as a certain understanding of the observed phenomena. In addition, in learning, linkage usually occurs slowly after several to many repetitions of the events, namely, in the form of Hebbian reinforcement of the synaptic connections between the neurons representing these events in the brain, whereas inference can occur upon a single presentation of the premises.

A simple way of testing whether animals can spontaneously make associations between perceptual and imaginary events is by confronting them with tasks in which information is missing. If animals, after seeing an experimenter baiting two boxes, one with food A and the other with food B, and then the experimenter eating food A, would they often or always choose the box with food B? If this task had never been given before so that the subject could not learn, would they still act correctly immediately? Would they understand that the box with the food that the experimenter did not eat (B) is still filled? Or if food is non-visibly hidden in one of two boxes, and then the experimenter is silently shaking one box, would the subjects understand that the other one is filled? Chimpanzees solved the task posed above without hesitation, not only after a phase of trial and error [15]. Later, also bonobos, orangutans, and gorillas were able to fill in the missing information in both the visual and auditory domains [16]. According to the authors, the monkeys showed “reasoning by exclusion”. Explanations based on fast learning and innate behavioural tendencies were ruled out with proper controls.

While everyday situations provoke the use of everyday heuristics, animals might be forced to use the full potential of their cognitive abilities when they must push their limits in abstract situations. In an abstract test for inference by exclusion on the touchscreen, parrots showed impressive abilities. Known for their unbridled curiosity, we tested kea parrots and Goffin’s cockatoos [17,18]. After being trained to perfection to discriminate between two images (S+, S−), the subjects were tested for their spontaneous response to four novel stimulus pairs. They contained the negative training image (S−) and a new image (S1), the positive training image (S+) and a second new image (S2), the image S1 and another new image (S3), and finally the second new image (S2) and a fourth new image (S4). Following the exclusion logic, the birds should choose S1, S+, S1, and S4. Choosing two times S1 is based on the pairing with S− in the first test (therefore it must be positive), and choosing S4 because it was paired with S2, which was paired in the second test with S+ and therefore assumed negative. Only sessions in which birds chose the correct image in all four tests (the random probability being two to the power of four, 1/16) were counted as successfully mastered. Most subjects of both parrot species solved the task.

Although only three out of six dogs have solved a similar task [19], one should not underestimate the cognitive abilities of at least a minority of dogs. The ability to infer what is correct by eliminating alternatives was famously shown by Ricoh, a border collie. After being trained by his owner to associate more than 200 everyday objects with words and to bring them up when the object was named, the dog was asked to bring a toy with a name he had never heard before. In fact, after an initial surprise at the new word, he brought the only object he did not know from the next room [20]. Since the dog had never perceived either the new word or the object before, his choice is consistent with the spontaneous formation of a new link. Taken together, dogs seem to have the potential to make inferences, to think in a kind of pre-logical manner by mentally connecting perceptual and imaginary events, and as Gifted Word Learners acquire novel object-label mappings even when the labels and objects are not presented simultaneously [21,22].

## 3. Are Dogs ‘Rational Imitators’?

In the field of animal social learning and culture, imitation occupies a special position. On the one hand, it has been considered a major engine of culture, language, and education [23]. On the other hand, it has been considered as one of the most advanced cognitive faculties: the observer acquires information about new techniques while at the same time drawing inferences about the efficiency of the observed methods, the constraints of the situation, and the intentions and goals of the model [24]. One reason why imitation has been explained in more cognitively advanced ways than by associative learning originates from the so-called “correspondence problem” [25]. For instance, facial expressions of a demonstrator cannot be compared with their own facial expressions—at least until one is in front of a mirror. Also, a quail would see something very different when it watches another quail pecking a lever and when it pecks the lever itself. So how could the observer quail know that its pecking behaviour is the same as the one demonstrated by the model? Some have therefore argued that associative learning cannot deal with this conundrum and that heavy cognitive machinery—such as supramodal mental representation or perspective-taking—is necessary to solve the correspondence problem [26].

Another test of imitation—one that was even explicitly characterized as ‘rational’—was performed with human infants. First, it was found that already 3- to 12-month-old infants interpret others’ behaviour as goal-directed [27] and, as a result, predict the most efficient action to achieve a goal, which was viewed as an example of teleological reasoning [28]. Even more surprising was the ‘rationality’ behind the imitation of 14-month-old children when exposed to two versions of demonstrations that differed in the situational constraints, that is, in the action possibilities of the demonstrator in the given situation [29]. When they watched a demonstrator illuminating a lightbox by leaning forward and touching its top with her forehead while her hands were occupied (pretending to be cold and holding a blanket wrapped around herself with both hands), they did not imitate the head action but used predominantly the more efficient method (touching the box with their hands) to achieve the goal. If, however, the less effective action (head) was demonstrated without any obvious reason to do so, they copied the demonstrated action. This finding shows that even children at this very early age identify what relevant information to retain and selectively imitate when observing others’ skills.

From a comparative cognition point of view, this surprising performance of young human children has provoked the question of whether non-human species would show an analogous capacity. One obvious candidate is the dog due to the proven ability to optimize their behaviour on the basis of efficiency and, even more importantly, because of their exceptional sensitivity to human-given communicative cues [30]. In an analogous experiment to the one with 14-month-old children, dogs watched a demonstrator dog pulling a rod with the paw instead of the preferred and more efficient mouth action. When the “inefficient” action was justified by the model’s carrying of a ball in her mouth, observer dogs used their preferred mouth action, but when no constraints could explain the demonstrator’s choice—the demonstrator dog had her mouth free—the observers imitated this action. Consequently, dogs, like children, demonstrated inferential selective imitation [31]. However, it remains a question whether this selectivity requires the attribution of mental states to others or whether it relies simply on the evaluation of the observable facts: the action, the goal state, and the situational constraints [28]. Anyhow, the parallels between dogs and children—and later also chimpanzees [32]—in terms of selective re-enactment of the demonstrated action seem to be influenced at least by the inference about efficiency. Interestingly, in both studies of children and chimpanzees, the authors claimed some understanding of the rationality of others’ intentional actions and their use when imitating others. In summary, dogs have shown several forms of social learning, from mimicry [33], contagion [34], emulation [35], and automatic imitation [36] up to those forms that have been considered human-unique, selective (or rational) imitation [24,31] and overimitation [37].

## 4. ‘Human-like’ Dogs?

In recent years, researchers have become increasingly interested in how dogs understand us humans, given that they show impressive abilities for interacting and communicating with us [38,39,40,41]. They are extremely attentive and interested in what humans are doing, have excellent learning abilities, can read subtle cues of human behaviour, and have extensive experience with different communicative situations. Such unusual sensitivity requires first and foremost a constant monitoring, i.e., looking at the human and being attentive to what he or she is doing [42]. Dogs monitor humans, especially their caregiver(s), not only to know what they are currently doing but also what they are interested in and therefore are doing next. They spontaneously focus attention on informative objects, such as eyes [43,44], decode our emotions [45,46], and are sensitive to human attentional states [47,48,49]—unlike wolves from puppy age on [50]. A much-researched case is the sensitivity to human social cues, especially using human pointing gestures to find hidden food [51,52,53]. Some have therefore concluded that the exceptional attentiveness towards humans has an innate component that was probably selected for during domestication [54]. Some researchers extended these proposals by hypothesizing that aspects of the social-cognitive abilities of dogs may show convergent evolution with those of humans, making them quite “human-like” [55,56,57].

Concerning their understanding of humans’ looking behaviour, researchers have designed tasks around two different behaviours—begging and obeying. When dogs were allowed to beg for food or for help from a human, they used the visibility of the humans’ eyes or the direction of their faces to decide who was attentive or not [49,58,59,60,61]. When dogs were given a command to either lie down [49] or to refrain from eating a piece of food in front of them [48], dogs behaved exactly in accordance with the degree of human attentiveness; the more attentive the commander was, the more they followed the command. This sensitivity to human attentiveness depends on more than one simple cue, like the open eyes of the human commander, but includes various human postures and attentional states [62] as well as features of objects that affect the human’s field of view, like visual barriers [63]. The visibility of the human or her eyes is not enough for dogs to decide whether she can see something or not. For example, dogs take into account whether forbidden food and the area around it are visible (illuminated) or not (in the dark) and whether the human commander is present or not [64]. They hesitated less to steal the food when it was in the dark, despite the human being illuminated. Obviously, they decided upon whether they themselves could be seen, not whether they could see the human commander.

## 5. Can Dogs Discriminate Between Guessers and Knowers?

Until a few years ago, the evidence for perspective-taking in dogs was at least ambiguous. When confronted with a task in which they had to decide whether a human person who forbade them to take food could see their stealing attempt or not in a situation where they themselves could not see the human (reaching with the paw into a transparent or opaque tunnel), they behaved indifferently [65]. The authors concluded that dogs, in contrast to chimpanzees in a similar task [66], are not able to overcome the egocentric perspective to conclude what the human can potentially see or not. In other words, they seem to rely on what they themselves can see when they assess what humans can see [54]. This conclusion was challenged when researchers applied the ‘Guesser–Knower’ task that was originally invented to test perspective taking abilities of chimpanzees [67]. This task requires the observer to appreciate the difference between their own and another’s line of sight [68], because subjects have to decide which of two potential human informants has seen a hidden event before or not. While a former study has not convincingly shown that dogs solve the task, especially because of a dramatic drop in performance after the first trial [69], a second study provided clear results in their favour. Maginnity & Grace [70] showed that dogs’ choices between two human informants were strongly influenced by cues related to the humans’ visual access to the food. They not only avoided trusting a human who looked at the ceiling or was absent during the hiding of the food but also preferred following the suggestion (pointing to a food container) of a human who covered her cheeks with her hands, rather than her eyes, during the food-hiding process.

**Figure 2:**
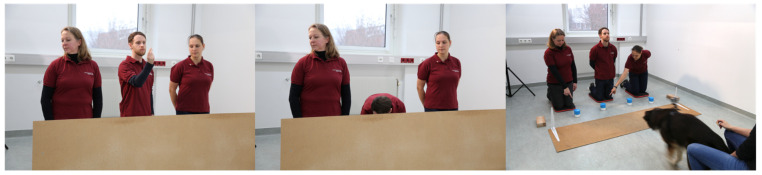
Still photos from the Looking Away condition in Catala et al. (2017) [71]. (**Left**): the Hider in the middle is showing a piece of sausage to the dog while the two informants are looking to the side in the same direction. (**Middle**): the Hider is hiding food behind the visual barrier. (**Right**): the dog is approaching the cups while the informants are each pointing to a different cup. Photo credit: Clever Dog Lab, Vetmeduni Vienna.

Although this study confirmed that dogs have a remarkable sensitivity to cues relating to humans’ attentional state, in this case, the visibility of the humans’ eyes and their gaze directions, it remained an open question whether the dogs’ assessment of a human’s knowledge can go beyond directly observable differences between the two informants. Therefore, we conducted a variant of the ‘Guesser–Knower’ task in which the human informants behaved identically [71]. Both informants looked in the same direction but at different positions in the room in relation to the centrally positioned Hider: kneeling on the left (Knower) or the right (Guesser) side of the Hider (see Figure 2). Therefore, they differed in what they could see from the baiting process. Importantly, the object of interest to the human (the hiding location) was not visible to the dogs; therefore, they could not simply use the eye–object line [60,72] but need to infer from the humans’ position (left or right of the Hider) what they could potentially see or not. In other words, they needed geometrical gaze following and a basic understanding that seeing leads to knowing (or guessing). After the hiding of the reward, the Knower and Guesser pointed to two different potential hiding locations, and the dogs were allowed to make a choice. In line with the geometrical gaze-following account, from the first trial, dogs preferred to follow the informant who had the hiding process in her field of vision (i.e., the Knower).

From this surprising result, the authors concluded that dogs are taking the informants’ position relative to the Hider into consideration. But it is also possible that the dogs have used subtle cues of the looking behaviour of the two informants. For instance, the Knower is—at least sometimes—following the hiding dynamically with her head and eyes, while the Guesser is looking away more statically. The active utilization of gaze cues from others has been demonstrated in cooperative (e.g., begging [58]) and competitive (e.g., stealing [62,64]) contexts and has been considered a pivotal advancement in the understanding of mental states, such as attention and intention. But it is still not what many have considered a mental state attribution.

This led the Viennese researchers to repeat the Guesser Looking Away condition with more dogs and with a more controlled looking behaviour of the informants. This time, both informants looked statically at the wall on the side of the Guesser. In addition, a Guesser Back Turning condition was applied—the Guesser turned her back to the hiding locations—to check whether this would help the dog to understand the Guesser’s inattentive behaviour. Actually, this was the case. Dogs followed the Knower’s pointing above chance level in the Guesser Back Turned condition but not the Guesser Looking Away condition (Lonardo et al., under review). It seems that the dogs followed the more attentive informant, but that the position in the room alone was not sufficient to develop a preference for the Knower. Therefore, it remained unclear whether the perspective-taking ability of dogs goes beyond direct evidence resulting from observable behavioural cues of others. Furthermore, dogs failed to show consistent behaviour across conditions and across time (with two repetitions in one year), and performance seemed to be strongly affected by procedural details. Perhaps this test is not a reliable tool to assess individual differences in the socio-cognitive ability of perspective-taking; other nonverbal tests are needed to ensure that conclusions drawn about perspective-taking in dogs are robust and interpretable.

## 6. Do Dogs Understand That Seeing Leads to Knowing?

In the past, several studies failed to show that dogs’ ‘mind-reading’ ability goes beyond the assessment of what humans *can* see by including an understanding of what humans *have* seen in the past and therefore *know* in the present. Although dogs were more likely to indicate the location of a hidden toy when the human helper had not witnessed the hiding event [73], an account in terms of different levels of arousal when owners reappeared could explain the results of this ‘ignorant helper’ test equally well [74]. And although the (single) subject in a further study did inform the helper in most cases in which the helper did not know where the object was hidden, rapid discrimination learning and extensive experience with his owner are possible explanations of this result [75]. One could argue that the ‘ignorant helper’ paradigm is not the most suitable one for challenging ‘mind-reading’ tasks because helping by showing is not the most natural behaviour of dogs. Therefore, Kaminski et al. [74] applied a fetching task in which dogs were asked by a human experimenter to bring the toy. While the dog could see the placement of two toys, the human experimenter could see the placement of only one (the experimenter was absent during the placement of the second). The dogs did not avoid fetching the toy, which the experimenter had not seen (and therefore could not have known about its existence), which provided a stronger case for dogs’ failure to infer what a human knows on the basis of what she has seen in the immediate past [74]. However, one might wonder why a dog should reflect on what the human commander has seen when it is so easy to obey the command (bringing a toy, not a specific toy).

### 6.1. Can Dogs Attribute Visual Access to Others Without Relying on Observable Visual Cues?

In the Guesser Looking Away condition of the Guesser–Knower task, the two informants were asked to look in the same direction to avoid obvious behavioural differences that help the dogs to understand differential perceptual access of others. Nonetheless, the dogs’ choice behaviour could have been based solely on responding to “observables”—stimuli that were physically present—rather than on mental states [76]. According to Heyes [77], dogs could, but they need not to, “understand” or have a “theory” about why the relationship between the position in the room and the knowledge of where food is hidden holds; they need not explain it to themselves with reference to what the “Knower” has “seen” and therefore “knows.” But if we exclude “mind-reading” (inferring others’ hidden mental states—intentions and beliefs—to predict behaviour), is the only alternative “behaviour reading” (observing external actions and stimulus–response patterns)? Perhaps not. Consequently, to overcome the “observables” problem by attributing visual access to others without relying on observable visual cues, it becomes essential to eliminate or control for all visually accessible differences that the perceiver could leverage to identify the Knower.

**Figure 3:**
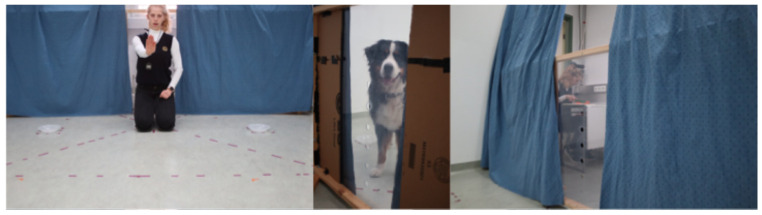
Still photos from Huber et al. (2025) [78]. (**Left**): the experimenter tells the dog (accompanied by a hand gesture) not to take the food. (**Middle**): the dog can see through the gap between the curtains the human chopping carrots. (**Right**): the experimenter is sitting behind the curtain and is chopping carrots. Photo credit: Clever Dog Lab, Vetmeduni Vienna.

One possibility to achieve this would be to let the dogs decide based on the remembered but not actual visual access to a human. In a recent experiment, dogs were unable to rely on (visually) observable features, such as open eyes, gaze orientation, or line of sight, because the human experimenter was absent in the test [78]. Dogs could steal food that they had previously been forbidden to take by a human experimenter at two spatially separated locations in the testing room (Figure 3). Only a chopping sound that the dogs heard in (only) one earlier exploration trial at the same time as when seeing a human experimenter chopping carrots could be used to infer the presence of the unseen human on the test. In this earlier exploration phase, they saw the chopping human from the position of one plate (the “seen plate”) in the room through a gap in a curtain, but not from any other position (especially not from the location of the other, unseen food plate). So, if the dogs remember from the pretest that they are not allowed to take the food, then infer the human presence based on the chopping sound (playback from a loudspeaker), and also transfer their prior visual experience of the location in the room where they had observed the human chopping carrots to the test situation, then they should choose the out-of-sight food source on the “unseen plate” in this test condition (“hidden stealing hypothesis”). As a control, we tested a second group of dogs exposed to street noise, a sound cue that should not elicit the expectation that the human was nearby. Thereby, we intended to create a condition in which the dogs would likely feel secure and, consequently, would exhibit no particular preference in choosing between the two food plates. This was exactly what we found. The majority of dogs (28 out of 36) that heard the chopping sound chose the unseen plate first, but only 20 out of 37 upon hearing the street noise.

There are, however, two possible explanations for this result, a cognitively richer one and a cognitively leaner one. In line with a Theory of Mind perspective, dogs have inferred from their previous experience in the exploration phase that the experimenter (whom they could only imagine), when sitting at the table chopping carrots, could see the “seen” plate and would thus be likely to interfere and remove the plate (as she did in the pre-test). This would be an altercentric attribution of visual access to an (unseen) human (“you can see me at the location where I have seen you previously”). But the dogs could also refrain from stealing food from a position where they could (egocentrically) see the experimenter who had forbidden them to take the food. They might have learned the heuristic that humans respond to their behaviour when they are in line of sight but not otherwise (“you will respond at the location where I can see you”). Therefore, they might avoid sight lines associated with the researcher being visible. Although the second, egocentric strategy—viewing the world strictly from one’s own viewpoint—is possible, the weight of evidence seems to lean towards the altercentric strategy—being influenced by, or centering on, another’s perspective. Dogs have stolen more food when they were hidden from a human’s view by a large barrier compared to a small one or one with a small window [63] or when the area surrounding forbidden food was illuminated, but not when in the dark [64]. Both studies suggest that dogs recognize the potential visibility of their bodies or their actions to the human and thus take into account the human’s visual access to the food while making their decision to steal it. Even more compelling, dogs had a preference for fetching one of two toys that was visible to the human, although both were visible to the dog [74]. Together, these studies suggest that dogs are sensitive to others’ visual access, even if that differs from their own; that is, they assume an altercentric perspective.

### 6.2. Can Dogs Discriminate Between True and False Beliefs?

Dogs might distinguish what they and others can see at the same time. But can they also distinguish what they and others have seen in the past and therefore know in the present (“what they believe”)? In the human literature, the benchmark test for this level of understanding is the ‘false belief’ task [79]. Here, one needs to take the perspective of others and not attribute one’s own knowledge to them, but to have the capacity to catch and understand someone else’s point of view, i.e., understand that another individual can hold a mistaken perspective [80]. However, as the original task was a verbal (explicit) one, it was not applicable to non-human animals. It became possible only when implicit false-belief tasks had been invented, in which young children who first failed elicited-response false-belief tasks demonstrated spontaneous false-belief understanding; for a review, see [81]. Also, the anticipatory looking behaviour of great apes suggested the ability to predict how others will act when they have a false belief about an object’s location [82].

Based on the human implicit false belief tasks, an analogous task for dogs was designed [83]. In this pre-registered ‘change of location’ test with 260 dogs, subjects could retrieve food from one of two opaque buckets after witnessing a misleading suggestion by a human informant who held either a true or a false belief about the location of food (between-subjects design). The dogs watched the initial hiding of food (by the Hider), its subsequent displacement by a second experimenter (the informant), and finally a misleading suggestion to the empty bucket by the informant. Unsurprisingly, most dogs (61.5%) were not fooled by the human and went straight to the baited (correct) container. But of those dogs that followed the human informant’s misleading suggestion to the empty container (38.5%), significantly more did so when the informant had a false belief (absent during the displacement) than when she had a true belief (absent before or after the displacement). Arguably, dogs responded to the communicator’s apparent knowledge about where food was, which differed according to the timing of her absence (whether she was absent or present during the displacement). However, while their differential reaction to the experimental manipulation suggests that dogs take human belief states into account, the dogs behaved in an opposite way to human infants and apes in similar paradigms [84,85]. This behavioural pattern might be explained by the way the dogs interpret the informant’s intention: dogs in the true belief group might have interpreted the informant’s misleading behaviour as deceitful (or driven by another, unknown intention), whereas dogs in the false belief group might have followed the informant’s wrong suggestion by attributing to her a false belief and consequently a “justified” mistake in good faith.

Despite this first evidence of the dogs’ ability to take human belief states into account, several important questions remain. On the one hand, the recent finding is at odds with the long-hold opinion that dogs are exquisite readers of our behaviour but not readers of our minds, e.g., [60], or have only a rudimentary Theory of Mind [86] or only Level 1 perspective taking (discerning what another individual can and cannot perceive from her own point of view, which differs from Level 2 perspective taking—forming a mental representation of the mental states of the other) [87,88], and therefore requires further confirmation. On the other hand, there are two puzzling aspects in those findings that warrant further tests. First, not all dogs behaved as described above. The most interesting deviation concerned dogs from FCI group 3, terriers (*N* = 50). They behaved like children and great apes; only a few followed the human informant with the false belief, significantly more followed the human informant with the true belief, and most followed their own knowledge about the final location of food. Perhaps this might be explained by their independent working style—terriers have been domesticated to find the hunter’s prey alone—while most other dogs in the study have been cooperative breeds [89]. Second, the post hoc explanation about why more dogs followed a human informant with a false belief rests on the assumption that they distinguished between different intentional actions [90]—one deceitful, the other one honest. Altogether, the performance of the dogs in the false belief test provided evidence of their ability to mentally detach themselves from the here and now and to consider not only obvious events but also the possibilities of what others intend and do next.

### 6.3. Can Dogs Understand the Other’s Perceptual States Independent of Their Own Perspective?

Apart from the slight preference for the false-belief communicator in contrast to humans, the study remained inconclusive in two respects: the limitation to the visual domain and the weak distinction between responding based on directly perceivable cues and additionally inferring others’ mental states. Concerning the latter, one could argue that the timing of the absence of the communicator (during versus before or after the food displacement) causes a perceivable difference between the false and true belief communicator. Furthermore, dogs can directly assess the attentional state of the human communicator by considering open eyes and gaze. Therefore, the researchers went one step further by designing a test in the auditory domain.

Arguably, if dogs are also able to conceal information in a modality other than the visual, this would show a deeper understanding of the other’s perceptual states [66,91]. Two studies indicated that dogs can use sound as a cue to locate food, even though they may not possess an understanding of the causal relationships involved in the noise production [92,93]. For instance, when forbidden to take food and then given a choice between a noisy container and a silent container from which to take it, dogs significantly preferred the silent container only when the human gatekeeper was not looking. When the human gatekeeper was looking, dogs showed no reliable pattern of approach [93]. This suggests dogs took into account the noise caused by their approach only when that noise could change what the experimenter knew about their actions. Furthermore, when dogs were unable to see the human in a task in which forbidden food was placed in a tunnel that they could retrieve by using their paw, they preferred a silent over a noisy approach [65]. Still, they could use the rule of thumb: “When I hear the noise, then the other hears it.” The question remains whether this is still an egocentric strategy or already a kind of experience-projection strategy.

In the new version of the change-of-location false-belief task, the researchers also investigated whether dogs are sensitive to a human communicator’s mental states about the location of food, but this time, the difference between the false and the true-belief communicator was not perceptible to the dogs. This time, only the dogs (*N* = 240) could see that food was hidden first in one opaque bucket (A) and then relocated to a second opaque bucket (B), but the communicator in both conditions turned away from the scene and showed his back to the dogs [94]. Both communicators could hear the hiding of food in bucket A due to the presence of bells on the lid of this bucket. But only the true belief communicator could also hear the hiding in B (bells ringing), while the relocation to B was made silent for the false communicator (silent bells without clappers). Importantly, both communicators behaved identically (crouching against the wall, then a misleading suggestion of A); therefore, the dogs need to infer what the communicator can hear or not and consequently are knowledgeable of where food is or not.

Dogs’ performances in this study led to somewhat ambiguous results [94]. On the one hand, the dogs’ responses did not differ significantly between the false and true belief conditions; on the other hand, they were not statistically different from those of the previous study [83]. Thus, either their auditory perspective-taking ability did not have a sufficiently large effect to be detected in this study, or it is less developed than their visual perspective-taking ability, or dogs are generally limited to egocentric strategies and direct behaviour cues by the other. It is also possible that the communicators’ strange behaviour—looking away from the hiding scenes and towards the wall—was interpreted by the dogs as inattentive or even uninterested, thereby losing any relevance of an informant. Only further studies that include a pretest phase that ensures that the later-tested dogs understand that the turning-away human is still attentive and aware of noisy actions behind them will bring us closer to a proper understanding of their Theory of Mind. In sum, Theory of Mind tests offer themselves as a promising window into the mind of the dog, as well as many other non-human species, but greater attention to the psychometric properties (construct validity and test–retest reliability) and to procedural details is necessary to make them mature tools for assessing perspective-taking abilities in non-verbal species.

## 7. Conclusions

The domestic dog is in an optimal position to offer insights into evolutionary processes of social and cognitive behaviour, and therefore has become an especially important species in the fields of cognitive ethology and comparative psychology [95]. One reason for this increased importance is the widely held hypothesis that domestication has had particularly important effects on dog social behaviour and cognition, altering the extent to which dogs accept and interact with humans as cooperative partners. The most interesting, though still disputed, feature of the process of domestication in dogs is its apparent functional convergence to human cognitive evolution, making dogs “human-like”, e.g., [55,56,57], although the suitability of dog domestication as a model for human social evolution is questionable [96]. Thus, one may wonder if this “human-likeliness” has also developed into the human capacity to weigh up possibilities, comparing alternatives in your mind, thinking ahead to future events, anticipating the consequences of your own actions, and even those of others, including heterospecific partners, so comprehensively that even the intentions behind them become apparent. If one considers practical rationality as incorporating these inferential capacities and of existing (also) outside of language and normativity, one may appreciate some behaviour of (some) pet dogs as being better explained in such terms than in purely associative or stimulus–response manners. Recently, researchers found some dogs (called Gifted Word Learner dogs) that even enter the realm of language-related cognition by spontaneously acquiring an extensive vocabulary of object labels without intentional training, during natural play interactions, by overhearing their owners’ interactions, and even when the labels and objects are not presented simultaneously [21,22,97,98]. Still, as a concluding remark, it is necessary to emphasize the fact that domestic (or pet) dogs represent only a very small proportion of dogs, and we do not yet know how strongly advanced (Popperian) cognition varies among domestic dogs. Nevertheless, the recent insights into the canid mind and the growing understanding of dog domestication have the potential to illuminate important transitions in the evolution of our own species, see [96].

## Data Availability

No new data were created or analyzed in this study.
